# Clinico-epidemiological Profile of Colorectal Cancer in Oncology Department of Tertiary Centre Hospital in Nepal: An Observational Study

**DOI:** 10.31729/jnma.9157

**Published:** 2025-07-31

**Authors:** Rita Kumari Mahaseth, Rashika Upadhyaya, Prakash Chandra Panjiyar, Neha Jha, Ujjwal Kumar Jha

**Affiliations:** 1 Department of Oncology, Nepal Medical College Teaching Hospital, Attarkhel, Kathmandu, Nepal; 2 Department of Nursing, Helping Hands Community Hospital, Chabahil, Kathmandu, Nepal; 3 Department of Forensic Medicine, Nepal Army Institute of Health Sciences, Sanobharyang, Kathmandu, Nepal; 4 Patan Academy of Health Sciences, Lagankhel, Lalitpur, Nepal.

**Keywords:** *clinico-epidemiological profile*, *colorectal cancer*, *tertiary centre hospital*

## Abstract

**Introduction:**

Colorectal cancer is a major global health concern with variable incidence and presentation across regions. The study was aimed to describe the clinic-epidemiological profile of colorectal cancer in a tertiary care hospital in Nepal.

**Methods:**

This observational cross-section study was conducted at a tertiary care hospital in Nepal from April 2021 to March 2024. Ethical clearance was obtained (Reference number: IRC/NMCTH/024/21). A total sampling method was used to include 69 patients with histologically confirmed colorectal cancer. Data were collected from patient records and analyzed using Statistical Package for the Social Sciences version 16.0. Point estimate at 95% Confidence Interval was calculated along with frequency and percentages for binary data, and mean and standard deviation for continuous data.

**Results:**

Out of 69 patients, 39 (56.52%) were male and 30 (43.48%) female. The most affected age group was 35-50 years, accounting for 26 (37.68%) patients. Rectum including recto-sigmoid colon was the most common tumor location in 38 (55.07%) cases. The most common presenting symptom was bowel habit change, seen in 46 (66.67%) patients. Lower anterior resection was performed in 26 (37.68%) cases. Histologically, moderately differentiated adenocarcinoma was the predominant type, found in 53 (76.81%) patients.

**Conclusions:**

Colorectal cancer in this study predominantly affected middle-aged males, with the rectum being the common site and moderately differentiated adenocarcinoma the main histological type.

## INTRODUCTION

Colorectal carcinoma is one of the most common health-related problems worldwide. According to GLOBOCAN 2020, colorectal cancer (CRC) is the third most commonly diagnosed cancer and the second leading cause of cancer deaths. Globally, 19.2 million people suffered from cancer, out of which 1.9 million had CRC, and over 0.93 million died due to it.^1^ Higher incidence rates are seen in Europe, Australia, and New Zealand (40.6 per 100000 males), while the lowest are in African and Southern Asian regions.^2^ CRC is more common in males, with the 61-70 years age group being most affected, and well-differentiated adenocarcinoma as the most common histological type.^3^ In Nepal, the age-standardized incidence and mortality rates were 5.8 and 4.8 per 100000 in 2018.^4^ While most cases occur above 45-50 years, 3% are found in those younger than 40.^5,6^ Multiple risk factors, both modifiable and non-modifiable, influence CRC development.^7^

There is limited literature from Nepal exploring the clinical and epidemiological characteristics of CRC. This study aims to assess the clinic-epidemiological characteristics, including incidence, demographic patterns, and clinical presentation, of CRC among patients at a tertiary care center in Nepal.

## METHODS

This was an observational cross-section study conducted at a tertiary care hospital in Nepal. The study included a total of 69 patients with histologically confirmed colorectal cancer who visited the oncology and pathology departments between April 2021 and March 2024. A total sampling method was used to select participants from the hospital records available during the study period.

Ethical clearance was obtained from the Institutional Review Committee (Reference number: IRC/NMCTH/024/21). Patient confidentiality was maintained throughout the study.

The inclusion criteria were patients across all age categories and both sexes with histologically verified colorectal adenocarcinoma diagnosis established through biopsy examination or surgical specimen analysis. Cases were enrolled irrespective of disease progression stage or therapeutic intervention received, including radical surgical procedures with curative intent, palliative management approaches, as well as chemotherapy and/or radiotherapy regimens. Exclusion parameters were restricted to subjects lacking tissue diagnosis confirmation of malignancy and those having anal canal malignancies, considering these constitute a separate disease category requiring distinct treatment protocols.

Data was collected by carefully reviewing records from the hospital. Trained assistants went through patient medical files, pathology reports, surgery notes, and oncology treatment records in a systematic manner. A standardised performa was made for this study to maintain uniformity in the information collected. This form included demographic details like age, gender, and residential address. It also covered clinical details such as the main symptoms at the time of presentation.

Details about the tumour were taken from the histopathology reports. These included the main site of the tumour (noted as rectum/rectosigmoid, ascending colon, transverse colon, or descending colon), the type of tumour tissue, how much the cells were differentiated (well, moderately, or poorly differentiated), and TNM staging, if available. Information about treatment included the type of surgery done (like right hemicolectomy, left hemicolectomy, or lower anterior resection), use of chemotherapy or radiotherapy before or after surgery, and palliative treatment given in advanced cases.

The data were analyzed based on various age group by making a category of greater than and lesser than 45 based on previous literature.^5,6^

Data entry and analysis were performed using Statistical Package for the Social Sciences version 16.0. Descriptive statistics were employed to summarize the study population characteristics. Continuous variables were expressed as means with standard deviations for normally distributed data or medians with interquartile ranges for non-normal distributions. Categorical variables were presented as frequencies and percentages.

## RESULTS

The age distribution of the study participants showed 6 (8.70%) were less than 35 years, 26 (37.68%) were between 35 and 50 years, 24 (34.78%) were between 50 and 65 years, and 13 (18.84%) were between 65 and 80 years with the mean age of 52.42 years ± 14.28 years . Among the participants, 39 (56.52%) were male and 30 (43.48%) were female (Table 1).

**Table 1:** Frequency Distribution of Children by Different Age Groups (n=182)

Age Group (years)	n(%)
Less than 35	6(8.70)
35-50	26(37.68)
50-65	24(34.78)
65-80	13(18.84)
Gender	
Male	39(56.52)
Female	30(43.48)

**Table 2:** Presenting Signs and Symptoms of Colorectal Cancer (n=69)

Signs and Symptoms	n(%)
Pain Abdomen	12(17.39)
Bowel/Bladder Habit Change	46(66.67)
Per Rectal Bleeding	39(56.52)
Palpable Abdominal/Rectal Mass	10(14.49)
Generalized Weakness	35(50.72)
Pallor	23(33.33)

**Table 3:** Tumor Location, Type of Surgery Performed, and Histological Type of Colorectal Cancer (n=69)

Item	n (%)
**Tumor Location**	
Rectum Including Recto-Sigmoid Colon	38 (55.07)
Ascending Colon	22 (31.88)
Transverse Colon	5 (7.25)
Descending Colon	4 (5.80)
**Type of Surgery Performed**	
Right Hemicolectomy	18 (26.09)
Left Hemicolectomy	8 (11.59)
Lower Anterior Resection (LAR)	26 (37.68)
Palliative Treatment	17 (24.64)
Histological Type	
Moderately Differentiated	53 (76.81)
Poorly Differentiated	16 (23.19)

**Table 4:** Association between age group and Preferred pain scale (n=182).

Characteristics	Less than 45 n (%)	More than 45 n (%)	Total n (%)
**Location of CRC**			
Rectum including recto-sigmoid colon	10 (14.49)	28 (40.58)	38 (55.07)
Ascending Colon	9 (13.04)	13 (18.84)	22 (31.88)
Descending Colon	1 (1.45)	3 (4.35)	4 (5.80)
Transverse Colon	2 (2.90)	3 (4.35)	5 (7.25)
**Types of Surgery Performed for CRC**			
Right Hemicolectomy	9 (13.04)	9 (13.04)	18 (26.09)
Left Hemicolectomy	3 (4.35)	5 (7.25)	8 (11.59)
Lower Anterior Resection (LAR)	10 (14.49)	16 (23.19)	26 (37.68)

Presenting signs and symptoms among the patients included pain abdomen in 12 (17.39%), bowel or bladder changes in 46 (66.67%), per rectal bleeding in 39 (56.52%), palpable abdominal or rectal mass in 10 (14.49%), generalized weakness in 35 (50.72%), and pallor in 23 (33.33%) patients. The mean duration from the appearance of first clinical symptoms to obtaining medical advice is 6.13 months. (Table 2).

The location of colorectal cancer was reported as rectum including recto-sigmoid colon in 38 (55.07%), ascending colon in 22 (31.88%), descending colon in 4 (5.80%), and transverse colon in 5 (7.25%) patients (Table 3). Types of surgery performed included right hemicolectomy in 18 (26.09%), left hemicolectomy in 8 (11.59%), and lower anterior resection (LAR) in 26 (37.68%) patients, while 17 (24.64%) received palliative treatment (Table 3). Histology type showed moderate differentiation in 53 (76.81%) and poor differentiation in 16 (23.19%) patients (Table 3).

Among the 69 patients with colorectal cancer, rectum including the recto-sigmoid colon was involved in 38 (55.07%) cases, with 10 (14.49%) patients aged less than 45 years and 28 (40.58%) patients aged more than 45 years. Ascending colon involvement was noted in 22 (31.88%) patients, with 9 (13.04%) in the less than 45 years group and 13 (18.84%) in the more than 45 years group. Descending colon was affected in 4 (5.80%) patients, comprising 1 (1.45%) in the younger group and 3 (4.35%) in the older group (Table 4).

## DISCUSSION

This observational cross-sectional study was conducted to assess the demographic characteristics and clinical profile of patients diagnosed with colorectal cancer, and to examine the association between these variables. Data were collected through patient records and files, encompassing a total of 69 individuals diagnosed with colorectal cancer.

The findings of the study revealed that colorectal cancer was most frequently diagnosed among individuals in the 35-50 years age group, accounting for 37.68% of the participants. This observation aligns with a prior study conducted at Menofia University, Egypt, between 2005 and 2010, which found that colorectal cancer was common among patients younger than 50 years of age.^8^ Similarly, a retrospective study at Mahavir Cancer Sansthan in Patna, conducted from January 2017 to December 2019, reported that approximately two-thirds of the patients were younger than 50 years, with an average age of 42 years.^9^ These consistent findings across different geographical locations suggest a rising incidence of early-onset colorectal cancer, warranting further investigation and potential adjustments in screening strategies.

In terms of gender distribution, this study found that 56.52% of the colorectal cancer cases were male. This finding is in agreement with the study from Menofia University, which also demonstrated a clear male predominance in colorectal cancer.^8^ Additionally, a cohort study conducted at Aljomhoria Hospital in Benghazi, which involved 152 patients, reported a male-to-female ratio of 1.2:1.0.^10^ The consistency in male predominance observed in multiple studies could reflect shared environmental, lifestyle, or genetic risk factors among male populations that merit further exploration.

With regard to clinical manifestations, the most frequently reported symptom among participants in this study was a change in bowel and bladder function, observed in 66.67% of the cases. In contrast, other studies have identified different predominant symptoms. For instance, both the cohort study at Aljomhoria Hospital and the retrospective analysis at Mahavir Cancer Sansthan indicated rectal bleeding and abdominal pain as the most common symptoms of colorectal cancer.^9,10^ This variation in symptomatology highlights the need for comprehensive clinical assessments and individualized diagnostic approaches. In addition to this, the mean duration of 6.13 months from the onset of symptoms to seeking medical advice in this study finding is similar to the study conducted in Tribhuvan University Teaching Hospital, Kathmandu where the mean duration from the onset of symptoms to seeking medical advice is 5.6 months.^11^ This finding indicates that, understanding this delay is crucial for designing interventions aimed at promoting timely medical consultations, improving health outcomes, and reducing the burden of the illness.

The current study identified the rectum, including the rectosigmoid colon, as the most frequently affected anatomical site, comprising 55.07% of the cases. This finding is consistent with a retrospective study conducted in the Medical Oncology Department in Blida, Algeria, where the recto-sigmoid region was similarly reported as the most commonly affected site, and adenocarcinoma as the most prevalent histological type.^12^ On the contrary, research from Menofia University found that colon cancer was more common than rectal cancer.^8^ A separate study from Misan Province in Iraq also reported that colorectal cancer is the most common gastrointestinal malignancy, primarily involving the left colon.^13^ These geographic variations in tumor location may reflect differences in dietary habits, genetic predispositions, or screening practices.

The type of surgical intervention varied according to the localization of the tumor. In the present study, lower anterior resection (LAR) was the most frequently performed procedure, accounting for 37.68% of cases. Conversely, in a study conducted at Tribhuvan University Teaching Hospital (TUTH) in Kathmandu, Nepal, spanning a three-year period from 2007 to 2010, right hemicolectomy was reported as the most commonly performed procedure, representing 23% of the cases.^14^ These differences in surgical approach emphasize the importance of tumor location and staging in guiding therapeutic decision making.

Regarding histopathological findings, 76.81% of the participants were diagnosed with moderately differentiated adenocarcinoma. This pattern is supported by a retrospective study conducted at Patan Hospital, Nepal, between 2016 and 2019, which also identified moderately differentiated adenocarcinoma as the predominant histological type among 36 patients.^15^ However, a differing trend was observed in a cohort study conducted at the Oncology Department of Aljomhoria Hospital and 7 October Hospital in Benghazi, where more than one-third of the patients had poorly differentiated adenocarcinoma.^10^ These differences in tumor differentiation may carry prognostic significance and highlight the need for histological evaluation in determining disease outcomes and treatment planning.

This study had several limitations that should be acknowledged. The sample size was relatively small, comprising only 69 patients from a single tertiary care center, which may limit the generalizability of the findings to the broader Nepalese population. Additionally, the observational cross-sectional design restricts the ability to infer causality or assess changes over time. Data were collected retrospectively from medical records, which may be subject to documentation bias and incomplete entries. Furthermore, molecular and genetic profiling, which are increasingly important in the management of colorectal cancer, were not included due to limited resources. These limitations highlight the need for larger, multi-center prospective studies incorporating advanced diagnostic modalities to obtain a more comprehensive understanding of colorectal cancer in Nepal.

## CONCLUSIONS

This study align with previous research that colorectal cancer in this population predominantly affects individuals aged 35-50 years, with a higher incidence in males and the rectum being the most common tumor site.

## Data Availability

The data are available from the corresponding author upon reasonable request.

## References

[1] GLOBOCAN 2020. (2024). Fact Sheet..

[2] Morgan E, Arnold M, Gini A (2023). Global Burden Of Colorectal Cancer In 2020 And 2040: Incidence And Mortality Estimates From GLOBOCAN. Gut..

[3] Magar CBP (2022). An Overview Of Colorectal Cancer In Tertiary Care Cancer Center Of Nepal.. Nepal J Cancer..

[4] World Health Organization. (2018). GLOBOCAN The Cancer Observatory: Nepal Factsheet..

[5] World Health Organization. (2024). GLOBOCAN: The Global Cancer Observatory: Nepal Factsheet 2022..

[6] Parkin D, Bray F, Ferlay J (2005). Global Cancer Statistics 2002.. CA Cancer J Clin..

[7] McDermott FT, Hughes ESR, Pihl E, Johnson WR, Price AB (1982). Local Recurrence After Partially Curative Resection For Rectal Cancer.. Br J Surg..

[8] Gohar SF, AlHassanin SA, El-Assal M, Hussein AM (2015). Clinico-Epidemiology Study Of Colorectal Cancer.. Nat Sci..

[9] Trivedi V, Chauhan R, Santosh S, Rani R, Singh U (2022). A Comparative Analysis Of Early-Onset Vs Late-Onset Rectal Cancer.. Ecancer medical science..

[10] Elzouki AN, Habel S, Alsoaeiti S, Abosedra A, Khan F (2014). Epidemiology Of Colorectal Carcinoma In Libya.. Avicenna J Med..

[11] Kansakar P, Singh Y (2012). Changing Trends Of Colorectal Carcinoma In Nepal.. Asian Pac J Cancer Prev..

[12] Bekouaci S, Smaili F (2019). Clinico-Epidemiological Profile Of Early-Onset Colorectal Cancer In Algeria.. Ann Oncol..

[13] Alhilfi HS, Almohammadawi KO, Alsaad RK (2019). Colorectal Cancer In Misan.. J Coloproctol (Rio J)..

[14] Lord L (2013). Colorectal Cancer At Tribhuvan University Hospital.. GUPEA..

[15] Paudyal S, Maharjan S (2019). Clinicopathological Profile In Nepal.. J Patan Acad Health Sci..

